# Hyper-Activated Pro-Inflammatory CD16^+^ Monocytes Correlate with the Severity of Liver Injury and Fibrosis in Patients with Chronic Hepatitis B

**DOI:** 10.1371/journal.pone.0017484

**Published:** 2011-03-01

**Authors:** Ji-Yuan Zhang, Zheng-Sheng Zou, Ang Huang, Zheng Zhang, Jun-Liang Fu, Xiang-Sheng Xu, Li-Ming Chen, Bao-Sen Li, Fu-Sheng Wang

**Affiliations:** 1 Research Center for Biological Therapy, Beijing 302 Hospital, Beijing, China; 2 Department of Infectious Diseases, Beijing 302 Hospital, Beijing, China; Instituto Butantan, Brazil

## Abstract

**Background:**

Extensive mononuclear cell infiltration is strongly correlated with liver damage in patients with chronic hepatitis B virus (CHB) infection. Macrophages and infiltrating monocytes also participate in the development of liver damage and fibrosis in animal models. However, little is known regarding the immunopathogenic role of peripheral blood monocytes and intrahepatic macrophages.

**Methodology/Principal Findings:**

The frequencies, phenotypes, and functions of peripheral blood and intrahepatic monocyte/macrophage subsets were analyzed in 110 HBeAg positive CHB patients, including 32 immune tolerant (IT) carriers and 78 immune activated (IA) patients. Liver biopsies from 20 IA patients undergoing diagnosis were collected for immunohistochemical analysis. IA patients displayed significant increases in peripheral blood monocytes and intrahepatic macrophages as well as CD16^+^ subsets, which were closely associated with serum alanine aminotransferase (ALT) levels and the liver histological activity index (HAI) scores. In addition, the increased CD16^+^ monocytes/macrophages expressed higher levels of the activation marker HLA-DR compared with CD16^−^ monocytes/macrophages. Furthermore, peripheral blood CD16^+^ monocytes preferentially released inflammatory cytokines and hold higher potency in inducing the expansion of Th17 cells. Of note, hepatic neutrophils also positively correlated with HAI scores.

**Conclusions:**

These distinct properties of monocyte/macrophage subpopulations participate in fostering the inflammatory microenvironment and liver damage in CHB patients and further represent a collaborative scenario among different cell types contributing to the pathogenesis of HBV-induced liver disease.

## Introduction

Chronic hepatitis B virus (HBV) infection is one of the most serious public health problems, with approximately 400 million HBV carriers worldwide, and 1–2 million people succumbing to HBV-induced liver cirrhosis and hepatocellular carcinoma each year, especially in China where approximately 22 million people suffer from chronic hepatitis B (CHB) [1]. HBV itself is non-cytopathic, but immune-mediated liver damage often occurs in patients with HBV infection and further contributes to disease progression [2]. The prevailing understanding has been that the HBV-specific CD8 T cells mainly contribute to killing virally infected hepatocytes [3]. Recent studies have shown that non-specific lymphocytes are significantly infiltrated in livers of CHB patients, such as dendritic cells (DCs), natural killer cells, Th17 cells and Treg cells, playing distinct roles in modulating the inflammatory process [4–7]. These findings, therefore, suggest that multiple types of immune cells may actively participate in HBV-associated liver pathogenesis [8]. Herein, understanding the reciprocal linkage between immune cells during chronic HBV infection is a prerequisite for developing effective treatment strategies for the disease.

As an important component of innate immunity, monocytes contribute directly to the immune defense against bacterial, protozoal, and fungal pathogens through strong activation of inflammatory cytokine responses [9]. The monocyte-related inflammatory activation can also be induced by some viral antigens [10]–[12]. In addition, cytokines such as IL-17 increased under inflammatory conditions effectively trigger the activation of monocytes [6]. Furthermore, different monocyte subsets with distinct phenotypic and functional characteristics are proposed to exist: classical CD14^high^CD16^−^ monocytes representing about 90% of circulating monocytes and the pro-inflammatory CD16^+^ monocytes. The latter can be further divided into two minor CD14^high^ CD16^+^ and CD14^low^ CD16^+^subpopulations [13]. These CD16^+^ monocytes selectively express surface antigens akin to tissue macrophages and produce higher levels of pro-inflammatory cytokines (e.g., TNF, IL-1, IL-6) compared to CD14^high^CD16^−^ monocytes. Increasing evidence shows that the increased CD16^+^ monocytes collaborating with CD14^high^CD16^−^ monocytes play an important role in shaping the inflammatory environment [14], [15]. More recently, elevated CD14^high^CD16^+^ monocytes were correlated with increased viral loads and decreased CD4^+^ T-cell counts in HIV infection [16]. However, very little is known about whether the CD16^+^ monocytes/macrophages contribute to the inflammatory environment in the livers of CHB patients.

Th17 cells with potent pro-inflammatory properties have gained considerable attention [17], [18]. Studies in other systems have found that the production of IL-17 by human Th17 cells critically depends on both the activation status and the anatomical location of accessory cells and seems to occur particularly in the presence of activated antigen-presenting cells such as monocytes or DC [19]–[21]. In a previous study, we found that Th17 cells increased with the severity of liver damage in CHB patients [6]. Notably, these Th17 cells in the liver spontaneously produced IL-17A. IL-17A can mobilize, recruit and activate neutrophils, leading to massive tissue inflammation and the progression of autoimmune disease and liver disease [22], [23]. However, whether monocytes/macrophages or their subsets can foster the inflammatory environment in livers of CHB patients, which further favor the activity of Th17 cells, remains largely unknown.

In the present study, we characterized monocytes/macrophages with their subsets, as well as neutrophils in CHB patients and found that these pathogenic cells were increased in their livers. Furthermore, these highly pathogenic cells may be linked to the inflammation in the livers of these CHB patients. These findings will facilitate the understanding of the mechanisms underlying the pathogenesis of liver damage and of hepatic immune cells during inflammation.

## Methods

### Patients

A total of 110 HBV-infected HBeAg positive subjects, including 78 immune activated (IA) patients and 32 immune tolerant (IT) carriers, were recruited in this study. All patients were diagnosed according to our previously described criteria [6], [7], [24]. Briefly, the IT group was defined as patients with high levels of circulating HBV DNA (>10^6^ IU/ml), but normal alanine aminotransferase (ALT) levels (normal range: <40 U/L). The IA group included patients with detectable circulating HBV DNA (>100 IU/ml) and elevated serum ALT levels (>40 U/L). None of the subjects with HBV infection included in the study had received antiviral therapy or immunosuppressive drugs within 6 months before sampling. Liver biopsies were collected from 20 IA patients, and the degree of hepatic inflammation was graded by the modified histological activity index (HAI) [25]. Briefly, two components, Grading (G) and Staging (S), were given in a numerical value ranged from 0 to 4: Grading was used to describe the intensity of necro-inflammatory activity, and Staging was a measure of fibrosis and architectural alteration in chronic hepatitis. Increased numerical value indicated the more severity of disease. Individuals with co-infections of HCV, hepatitis D virus, and HIV infections were excluded from enrollment. The study protocol was approved by Beijing 302 Hospital Research Ethnics Committee, and written informed consent was obtained from all participants. Baseline clinical data are shown in Table 1. For comparison, 38 uninfected healthy controls (HCs) were age and sex matched to the enrolled patient as controls.

**Table 1:** Clinical characteristics of the populations enrolled in the study.

Group	HC	IT	IA
Case	38	32	78
Sex (male/female)	20/18	18/14	40/38
Age (years)	31 (18–42)	30 (18–44)	35 (18–49)
ALT (U/L)	21 (10–37)	27 (13–40)	144 (43–1656) *
HBV DNA (IU/ml)	ND	1.86×108(1.77×106–2.198×109)	2.3×107(110–2.53×109) *
Cases with liver biopsy	0	0	20
Inflammation Score			
G1	0	0	9
G2	0	0	7
G3	0	0	3
G4	0	0	1
Fibrosis Score			
S1	0	0	12
S2	0	0	3
S3	0	0	4
S4	0	0	1

Data are shown as median and range. HC: healthy control; IT: immune-tolerant carriers with HBV infection; IA: immune-active patients with HBV infection. ND: not determined.

*P<0.05 vs. IT subjects.

### Flow cytometric analysis

All antibodies were purchased from BD Biosciences (San Jose, CA, USA) except for phycoerythrin (PE)-conjugated anti-human IL-17A.

For surface marker staining, fresh heparinized peripheral blood (100 µl) was labeled with the following monoclonal antibodies: allophycocyanin (APC)-conjugated anti-human CD14, fluorescein isothiocyanate (FITC)-conjugated anti-human CD16 and peridin chlorophyll protein (PerCP)-conjugated HLA-DR. Matched isotype antibodies were used as negative controls. After incubation for 30 min at 4°C in the dark, FACS™ lysing solution (BD PharMingen) was added to lyse the red blood cells.

For intracellular cytokine staining, fresh heparinized peripheral blood (200 µl) was incubated in 800 µl RPMI 1640 medium supplemented with 10% fetal calf serum for 6 h as previously described [6]. Monensin (0.4 µM, BD PharMingen) was added during the first hour of incubation. After incubation, the blood was washed with PBS and stained with APC-conjugated anti-human CD14, FITC-conjugated anti-human CD16 and PerCP-conjugated anti-human HLA-DR for 30 min at 4°C in the dark. Subsequently, the red blood cells were lysed with FACS™ lysing solution, and the remaining cells were further permeabilized, stained with the corresponding intracellular antibody, fixed, and analyzed using FACSCalibur and FlowJo software (Tristar, USA) as previously described [14].

### Cell isolation

Peripheral blood mononuclear cells (PBMCs) were isolated by Ficoll–Hypaque (Pharmacia, Uppsala, Sweden) density gradient centrifugation from heparinized blood. Monocyte counts of these patients were determined by an automated hematology analyzer (KX-21N, Sysmex) according to our previously reported protocols [26].

Liver biopsy specimens were homogenized for isolation of intrahepatic leukocytes (LILs) according to our previously described protocols [7], and then LILs were washed in PBS and resuspended in RPMI 1640 with 10% FCS for direct staining with the antibodies.

### CFSE-based proliferation assay and stimulation

Carboxyfluorescein succinimidyl ester (CFSE)-based proliferation assays were performed according to our previously described protocols [26]. Cells were then stimulated with anti-human CD3 and CD28 (both 1 µg/ml) or HBV core antigen (5 µg/ml) in the presence of human rIL1β, rIL6 and rIL23 (each eBioscience; 10 ng/ml) in a 24-well plate. On day 6, the cells were re-stimulated with phorbol 12-myristate 13-acetate (PMA, Sigma-Aldrich; 300 ng/ml) and ionomycin (Sigma-Aldrich; 1 µg/ml) for 5 h in the presence of monensin. Cells were then stained with APC-conjugated anti-human CD3 and PerCP-conjugated anti-human CD8, and further permeabilized and stained with intracellular antibody PE-conjugated anti-human IL-17 [6].

### Immunohistochemistry

Paraffin-embedded, formalin-fixed liver tissue (5 µm) was incubated with anti-CD68 (a marker for monocyes and macrophage) or anti-myeloperoxidase (MPO, specific for neutrophils) antibody overnight at 4°C after blocking endogenous peroxidase activity with 0.3% H_2_O_2_. 3-amino-9-ethyl-carbazole (red color) was used as the substrate followed by counterstaining with hematoxylin for single staining according to previously described protocols [5]–[7].

### Serum HBV DNA assays

The serum HBV DNA assays was performed according to our recent description [5]–[7], [26]. The cut-off value was 100 IU/ml.

### Statistical analysis

All data were analyzed using SPSS version 13.0 for Windows software (SPSS Inc., Chicago, IL). Comparisons between various individuals were performed using the Mann-Whitney *U* test. Comparison between values within the same individual was performed using the Wilcoxon matched pairs *T* test. For all tests, two-sided *P*<0.05 was considered to be significant.

## Results

### Peripheral monocytes and hepatic monocytes/macrophages are significantly increased in IA patients

**Figure 1:**
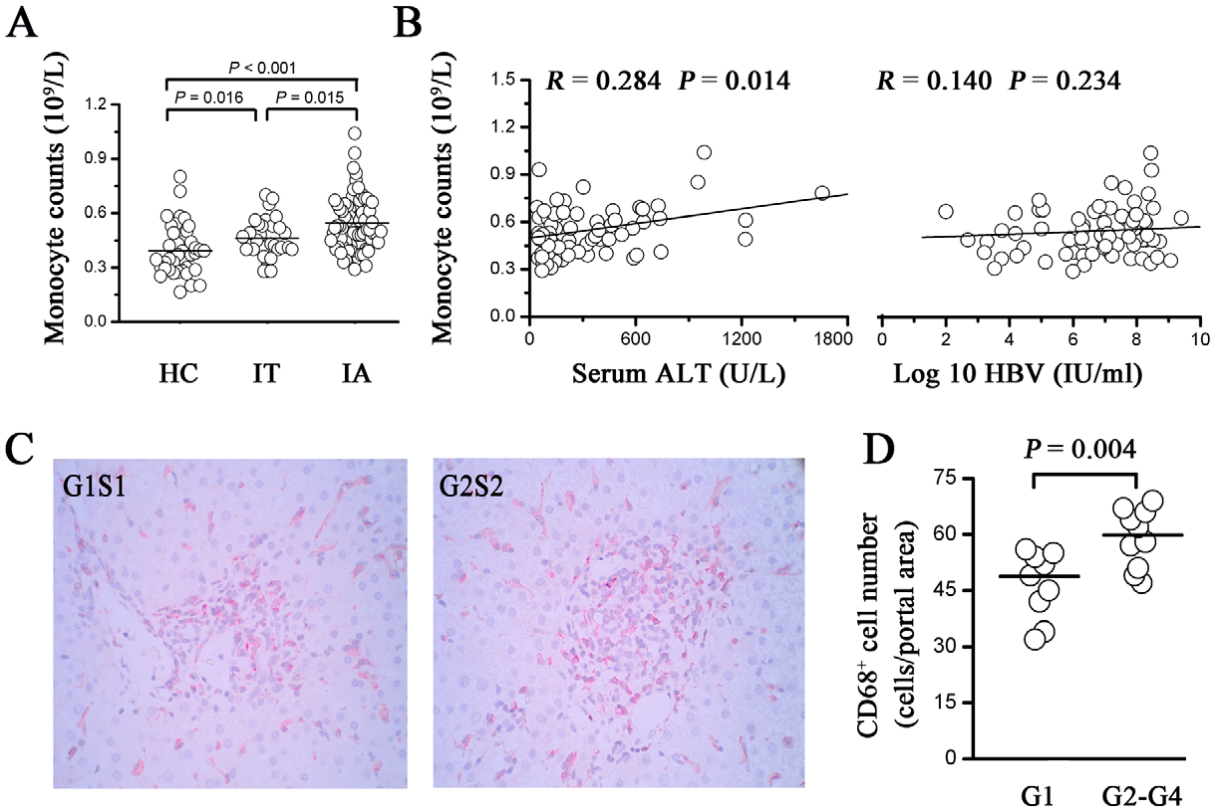
Increased monocytes in peripheral blood and liver positively correlate with liver injury in IA patients. (A) Absolute counts of the peripheral blood monocytes from IT (n = 32), IA (n = 78) patients and healthy controls (n = 38). Each dot represents the value from one individual. *P* values are shown. (B) The absolute numbers of peripheral blood monocytes was significantly correlated with serum ALT levels but not with HBV DNA. The solid line represents the linear growth trend and *r*, the correlative coefficient. *P* values are shown. (C) Representative immunohistochemical staining of CD68 in specimens from IA patients. An IA patient with HAI G2S2 scores has higher CD68^+^ cell density in liver, compared to that of a patient with HAI G1S1 scores. (D) Numbers of CD68^+^ cells in portal area are shown in IA patients with various degrees of liver HAI scores. Horizontal bars represent the median CD68^+^ cells. ALT, alanine aminotransferase; HAI, histological activity index; IT, Immune tolerant carriers; IA, immune activated patients.

We first analyzed the peripheral monocytes in CHB patients and healthy controls. As shown in Figure 1A, the absolute numbers of peripheral monocytes were increased in CHB patients, of whom the IA patients had the highest levels compared with IT patients and healthy controls (0.55±0.17, 0.46±0.11 and 0.40±0.14; 10^9^/L; for IA, IT patients and healthy controls).

Next, we analyzed the correlations between the absolute number of monocytes and serum ALT levels and plasma HBV DNA load in these IA patients. A significant positive correlation was observed between monocyte number and serum ALT levels (*r* = 0.284, *P* = 0.014), but no correlation was observed between monocyte numbers and serum HBV DNA loads (*r* = 0.140, *P* = 0.234; Figure 1B) in these IA subjects. We next examined the distribution of CD68^+^ cells in the livers of CHB patients. As shown in Figure 1C, an IA patient with HAI G2S2 that had a higher CD68^+^ cell density in the liver than did a patient with HAI G1S1. Because CD68 molecule was widely expressed on Kupffer cells in lobular area, the number of CD68^+^ cells in portal area was defined as the macrophages for quantitative analysis. We found the numbers of CD68^+^ cells in the portal area were markedly accumulated in livers of CHB patients with high HAI scores (Figure 1D).

### Pro-inflammatory CD16^+^ monocytes are significantly increased in peripheral blood of IA patients

**Figure 2:**
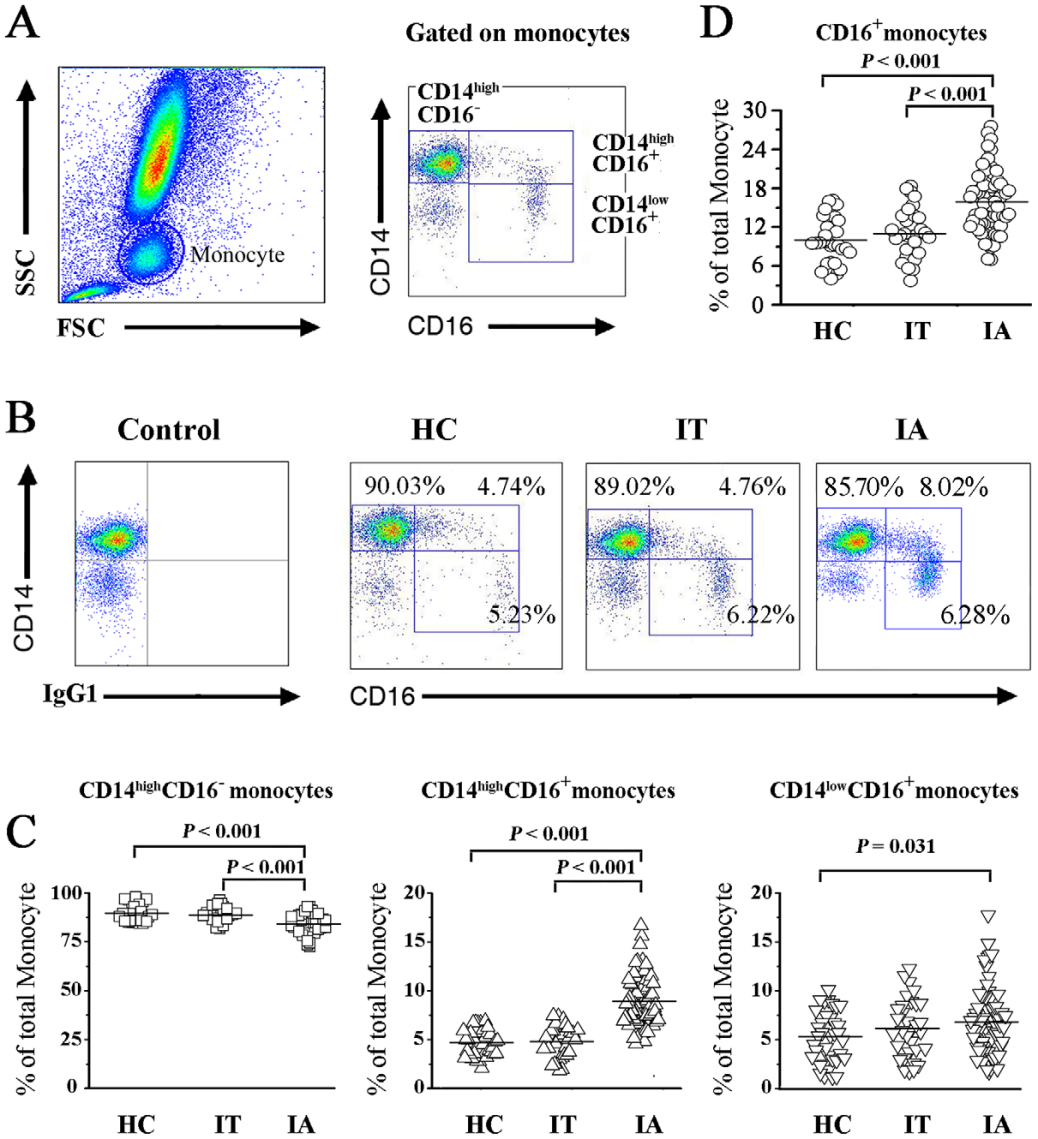
Peripheral CD16^+^ monocyte frequencies are significantly increased in IA patients. (A) Monocyte subsets in whole blood were identified based on forward and side scatter (left panel) and CD14/CD16 expression patterns (right panel). Monocytes were further divided into the CD14^high^CD16^−^ subset and a major CD16^+^subsets; the latter were further divided into CD14^high^CD16^+^ and CD14^low^CD16^+^ subsets for analysis. (B) Representative dot plots of three monocyte subsets in IT, IA patients and healthy controls. Values in the quadrants indicate the proportions of monocyte subsets. (C) Individual values of the three monocyte subsets in IT, IA patients and healthy controls. *P* values are shown. Horizontal bars represent the median proportions of each monocyte subset. (D) Individual values of major CD16^+^ monocyte subsets in IT, IA patients and healthy controls. *P* values are shown. Horizontal bars represent the median proportions of the CD16^+^ monocyte subsets. IT, Immune tolerant carriers; IA, immune activated patients.

Monocytes are generally categorized into the CD14^high^ CD16^−^ subset and the major CD16^+^ subsets, the latter being further divided into CD14^high^CD16^+^ and CD14^low^CD16^+^ subsets (Figure 2A). As shown in Figure 2B, all subjects clearly displayed 3 subsets of peripheral blood monocytes. Notably, the distribution of these subsets in HBV-infected subjects differed from that of HC subjects.

IA patients exhibited a decreased percentage of the CD14^high^ CD16^−^ monocyte subset and increased percentage of CD14^high^ CD16^+^ monocytes as compared to HC and IT patients (*P*<0.05, Figure 2C). By contrast, there were no significant differences in the three monocytes subsets between IT patients and HC subjects. A slight increase in the frequency of the CD14^low^ CD16^+^ monocytes was observed in IA patients versus HC subjects (*P*<0.05). Thus, as shown in Figure 2D, the proportion of CD16^+^ monocytes were significantly increased in the peripheral blood of IA patients.

### Increased pro-inflammatory CD16^+^ monocytes in peripheral blood and CD16^+^ macrophages in liver positively correlate with liver inflammatory damage in IA patients

**Figure 3:**
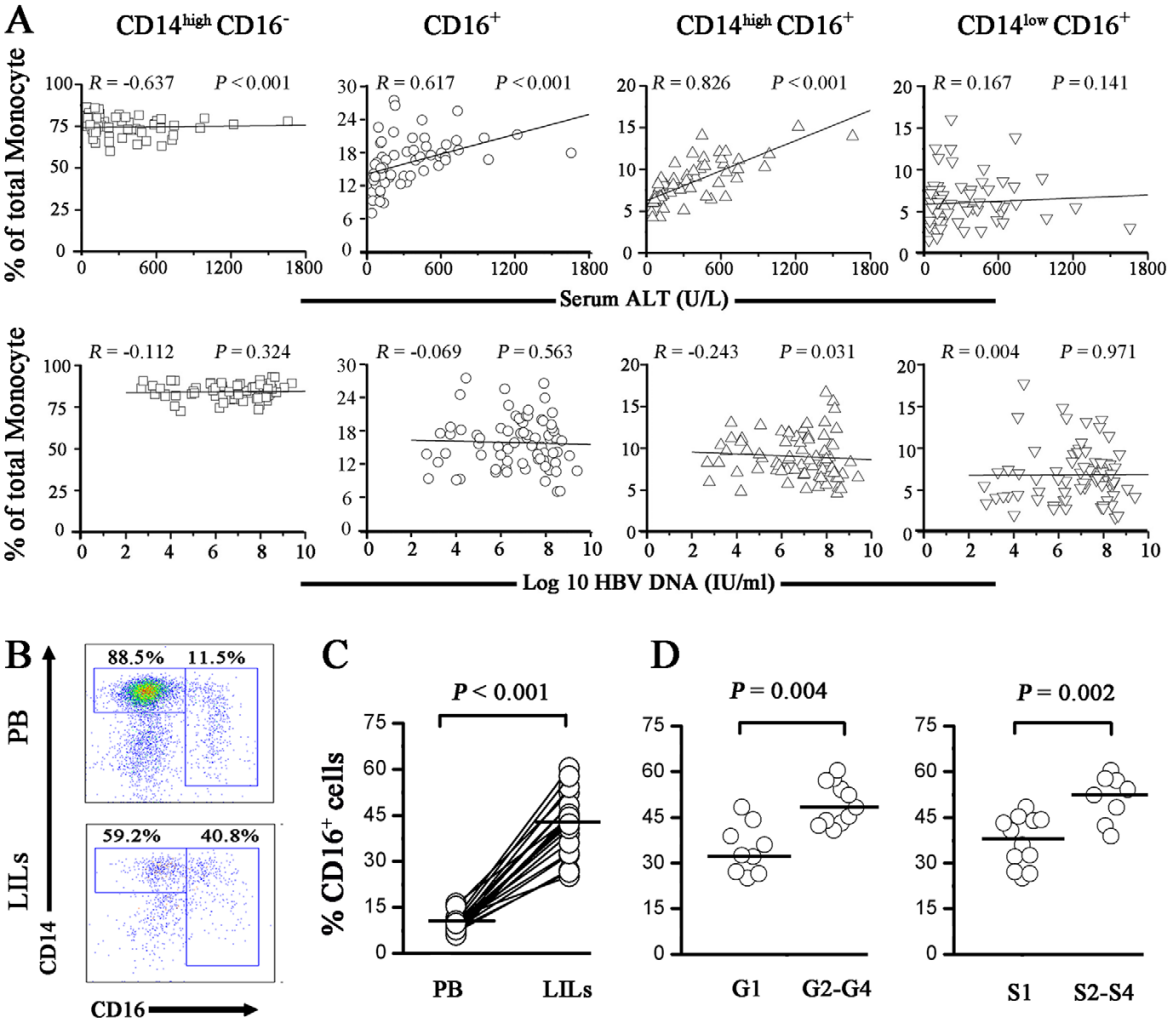
Increased CD16^+^ monocytes in peripheral blood and liver positively correlate with liver injury in IA patients. (A) The major CD16^+^ monocytes and CD14^high^CD16^+^ monocyte subsets were significantly correlelated with serum ALT levels, but not with HBV DNA. The solid line represents the linear growth trend and *r*, the correlative coefficient. *P* values are shown. (B) Representative dot plots of CD14 and CD16 staining in monocytes from peripheral blood and LILs isolated from the the same IA patient are shown. Values in the upper-left and upper-right quadrants represent the percentages of CD14^high^CD16^−^ monocytes and CD16^+^ monocytes, respectively. (C) Individual percentages of hepatic and peripheral monocytes that express CD16 from the IA patients. Each dot represents one individual. *P* values are shown. (D) IA patients with higher HAI scores (G and S) have a higher percentage of monocytes expressing CD16 in their livers, compared to patients with lower HAI scores. *P* values are shown. ALT, alanine aminotransferase; HAI, histological activity index; LILs, intrahepatic leukocytes.

We next analyzed the correlation between the percentage of monocyte subsets and serum ALT and plasma HBV DNA loads in these IA patients (Figure 3A). There was a significant positive correlation between CD16^+^monocytes frequency and serum ALT levels (*r* = 0.617, *P*<0.001), but no correlation with serum HBV DNA load in IA subjects was observed. Interestingly, we observed some significant positive correlations between CD14^high^ CD16^+^monocytes frequency and serum ALT levels (*r* = 0.826, *P*<0.001) and a negative correlation with serum HBV DNA load (*r* = 0.243, *P* = 0.031) in IA subjects. In contrast, no correlations were found between CD14^low^CD16^+^monocytes and these clinical markers.

We further dissected the populations of liver-infiltrating macrophages in LILs from IA patients. Figure 3B shows that these liver-derived macrophages had increased expression of CD16 and that these CD16^+^ macrophages are present at significantly increased numbers in LILs compared with peripheral blood (Figure 3C). We found that CHB patients with high HAI grading (G2–G4) (n = 11) had a greater proportion of CD16^+^ macrophages than did CHB patients with low HAI grading (G1, n = 9). Strikingly, we also found that CHB patients with high HAI staging (S2–S4) (n = 8) had a greater proportion of CD16^+^ macrophages than did CHB patients with low HAI scores (S1, n = 12) (Figure 3D).

These data suggest that the frequency of peripheral CD16^+^ monocytes is closely associated with liver injury as indicated by the correlation with ALT levels and that the expression of CD16 on liver macrophages is associated with liver injury as indicated by the correlation with HAI scores in IA patients.

### Activated pro-inflammatory CD16^+^ monocytes induce Th17 cell proliferation

**Figure 4:**
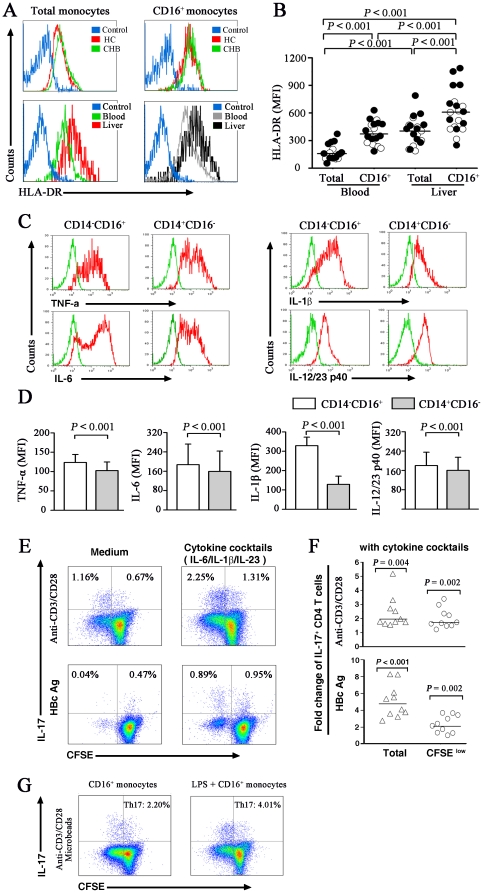
Hyper-activated CD16^+^ monocytes preferentially produce Th17-related cytokines. (A) Histogram plots of HLA-DR expression on monocytes and CD16^+^ monocytes from circulating monocytes (blood) and liver-infiltrating monocytes (liver) in a representative IA individual. (B) The MFI values of HLA-DR expressed on monocytes and CD16^+^ monocytes from CHB patients in each group. Each open dot represents one subject with lower HAI scores and each solid dot represents patients with higher HAI scores. (C) Representative histogram plots of TNF-α, IL-6, IL-1β, IL-12/23 p40 expression (red line histograms) or isotype control (green line histograms) on CD14^+^ CD16^−^ monocytes and CD16^+^ monocytes in an IA subject. (D) Data shown are representative of ten independently performed experiments from IA subjects. (E) Representative expansion of Th17 cells with the cytokine cocktail (IL-6, IL-1β and IL-23) and TCR-stimulation. Values in the upper left quadrant indicate the percentages of CFSE^low^ Th17 cells. (F) Individual values of the fold changes in the percentage of total and CFSE^low^ Th17 cells. Horizontal bars indicate the median fold changes. (G) Representative expansion of Th17 cells with activated CD16^+^ monocytes in HC subjects (n = 4). Purified CD4^+^ T cells from HCs were cocultured with autologous CD16^+^ monocytes and anti-CD3/CD28 microbeads in the absence or presence of LPS. After 4 days, cells were stimulated for 6 h with PMA/ionomycin and Golgi-stop (during the last 5 h), and IL-17 expression and production were determined by ICC staining. Values in the upper quadrant represent the percentage of Th17 cells. MFI, mean fluorescence intensity; HAI, histological activity index; IA, immune activated patients; CFSE, Carboxyfluorescein succinimidyl ester.

The production of IL-17 by human Th17 cells depends on the activation status and the anatomical location of monocytes [19]–[21]. We then identified the phenotypic features of liver-derived monocytes by using flow cytometry. The results showed that the liver-derived monocytes expressed higher levels of HLA-DR compared with that from peripheral blood indicated by mean fluorescence intensities (MFI) (Figure 4A). Notably, liver-derived CD16^+^ monocytes exhibited higher levels of HLA-DR compared with its counterpart in peripheral blood (Figure 4B). Further analysis showed that CD16^+^ monocytes in liver from CHB patients with high HAI grading (G2-G4, solid dot, HLA-DR MFI: 692.79±268.05) seemed to have slightly increased MFI values of HLA-DR compared with CHB patients with low HAI grading (G1, open dot, HLA-DR MFI: 528.44±135.14, *P* = 0.049).

Monocytes from CHB patients secreted increased cytokines, including IL-1β, IL-6, IL-23, and TNF, which have been shown to regulate the development of Th17 cells [19]–[21]. We first examined the production of these pro-inflammatory cytokines in subsets of monocytes under LPS stimulation *in vitro*. As shown in Figure 4C, monocytes produced large quantities of these cytokines, and there was no difference in the frequencies of cytokine-producing monocytes within the monocyte subsets in the peripheral blood. However, CD16^+^ monocytes seemed to have increased secretion of TNF, IL-6, IL-1β and IL-12/IL-23 p40 than did CD14^high^CD16^−^ monocytes, indicated by mean fluorescence intensities (MFI) of the labeled cells in these CHB patients (Figure 4D).

TNF is one of the final mediators of destructive activity on hepatic cells, but blockade of TNF has no effect on the expansion of Th17 cells [26]. In this section, we investigated the response of Th17 cells under TCR-stimulation (anti-CD3/CD28 or HBV antigen) together with exogenously added IL-1β, IL-6 and IL-23 to mimic the inflammatory environment in the livers of CHB patients. The ratios of the numbers of IL-17-producing cells and proliferating CFSE^low^ Th17 cells compared to those in the absence of the cytokine cocktail (considered as 1) was calculated for each response, and the fold changes are shown in Figure 4E–4F. We found that TCR-stimulation alone triggered only a low level of Th17 cell proliferation, whereas the addition of the cytokine cocktail, along with TCR-stimulation, profoundly induced proliferation of Th17 cells and the accumulation of CFSE^low^ Th17 cells among these CD4 T cells. In addition, an increased percentage of IL-17^+^ cells were found when purified CD4^+^ T cells were stimulated in the presence of LPS-activated CD16^+^ monocytes (Figure 4G).

### Neutrophils are reduced in peripheral blood, but accumulated in the livers of in IA patients

**Figure 5:**
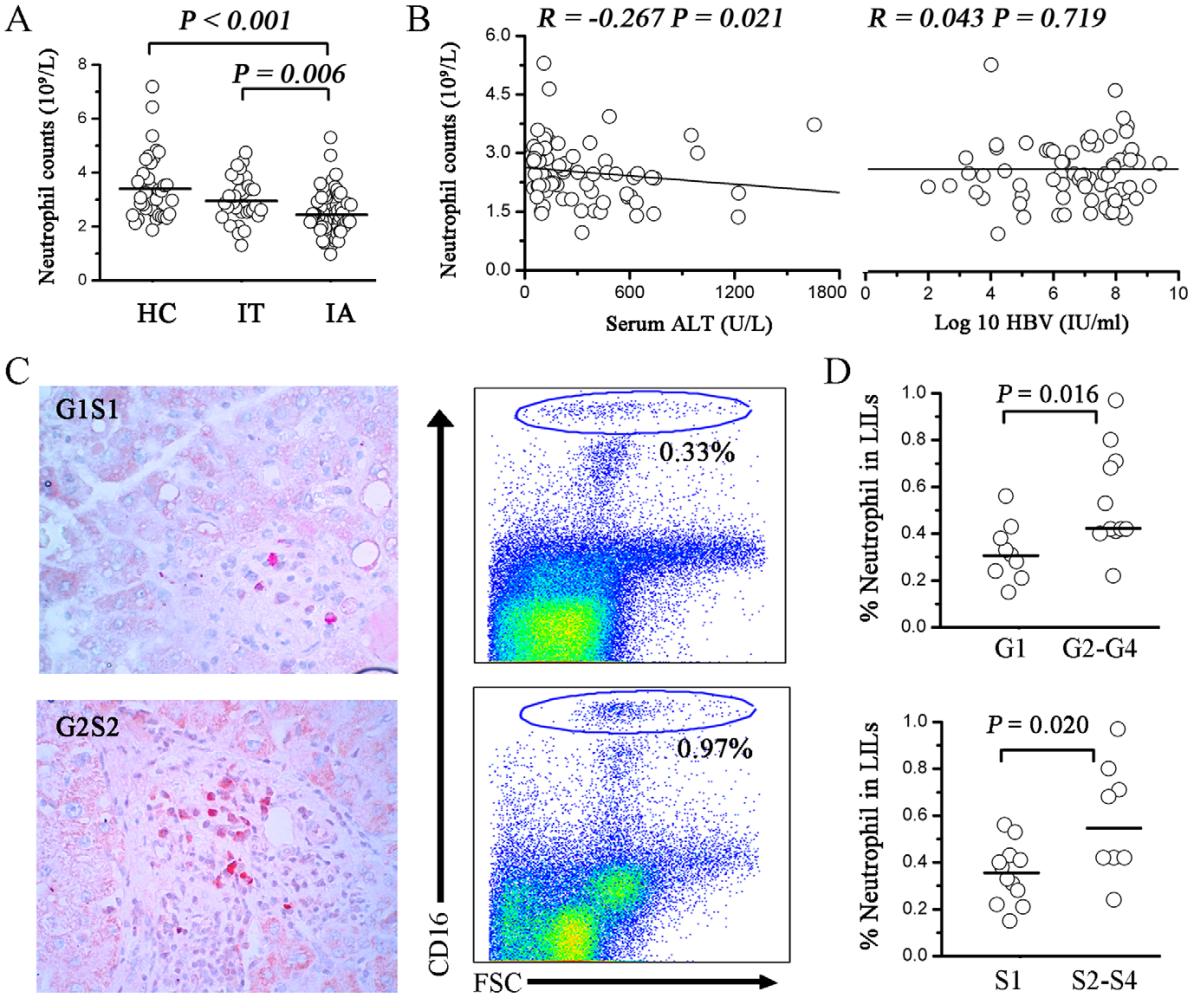
Increased neutrophils in liver *in situ* positively correlate with liver injury in IA patients. (A) Absolute counts of peripheral blood neutrophils from IT (n = 32), IA (n = 78) patients and healthy controls (n = 38). Each dot represents one individual. *P* values are shown. (B) The absolute number of neutrophils significantly negatively correlated with the serum ALT levels, but not with HBV DNA. The solid line represents the linear growth trend and *r*, the correlative coefficient. *P* values are shown. (C) Immunohistochemical staining for MPO (specific for neutrophils) in the portal area in IA patients with HAI scores (G1S1 and G2S2) (400×). A G2S2 patient had a higher percentage of neutrophils in LILs compared to a G1S1 patient. Values in the upper right indicate the percentages of neutrophils in LILs. (D) IA patients with higher HAI scores (G and S) have a higher percentage of liver neutrophils in the portal area, compared to patients with lower HAI scores. Each dot represents one individual. *P* values are shown. Horizontal bars represent the median proportions of liver neutrophils. IT, Immune tolerant carriers; IA, immune activated patients. HAI, histological activity index; LILs, intrahepatic leukocytes.

We observed that neutrophils in the peripheral blood from CHB patients were significantly decreased compared with those of healthy controls, and they correlated with disease stage in CHB patients. As shown in Figure 5A, a significant decrease of absolute numbers of neutrophils was shown in IA patients versus IT patients and healthy controls (2.51±0.77, 2.97±0.82 versus 3.42±1.19; 10^9^/L).

Next, we analyzed the correlations between absolute numbers of neutrophils and serum ALT levels and plasma HBV DNA load in these IA patients. The number of neutrophils were significantly positively correlated with serum ALT levels (*r* = −0.267, *P* = 0.021), but not with serum HBV DNA load (*r* = 0.043, *P* = 0.719; Figure 5B) in these IA subjects. Strikingly, more MPO^+^ cells were found to be infiltrated in the livers of patients with a G2 score than those of patients with G1 scores. Similar results were also obtained when we measured the percentages of neutrophils in LILs (Figure 5C) which progressively increased in patients with HAI scores: more neutrophils infiltrated the livers of patients with higher G and S scores (Figure 5D).

## Discussion

Non-HBV-specific infiltration of inflammatory cells into the liver is likely responsible for liver pathology during chronic HBV infection in humans; however, very little information available is known about the compartmentalization of cell types within the affected liver, let alone about the crosstalk among these cells. Here, we for the first time assessed the quantities, percentages and cytokine profiles of peripheral and intrahepatic monocyte/macrophage subsets in CHB patients and documented a significant increase in peripheral monocytes and intrahepatic macrophages as well as CD16^+^ subsets, which were closely associated with the liver inflammation. Moreover, these increased CD16^+^ monocytes preferentially released inflammatory cytokines, a process likely involved in the induction of liver damage and expansion of Th17 cells. Furthermore, the accumulated hepatic neutrophils were associated with liver injury. These distinct properties of monocyte/macrophage subpopulations as well as highly pathogenic neutrophils may further represent a collaborative scenario among different types of cells leading to the pathogenesis of HBV-induced liver disease.

Monocytes/macrophages are involved in various liver injuries; however, to our knowledge, different monocyte subsets from peripheral blood and liver have not been previously associated liver damage in CHB patients. As such, we first characterized absolute numbers of monocytes in a cohort of CHB patients, and found that the monocytes were increased in peripheral blood of IA over IT patients. This finding was further supported by the observation of more intrahepatic monocytes in IA patients. The present study demonstrated that the increased CD16^+^ pro-inflammatory subset of monocytes in CHB patients is associated with liver injury. There are three observations to support this notion. First, the CD16^+^ subset frequencies in peripheral blood were positively correlated with serum ALT levels in IA patients. Notably, the increased CD16^+^ subset frequencies were mainly due to an increased in the absolute number of these cells. In addition, patients with higher HAI scores had more CD16^+^ monocytes/macrophages in the liver *in situ* than did patients with lower HAI scores from analysis of the liver biopsies. In combination with previous report in chronic inflammatory liver disease that CD16^+^ monocytes were recruited by a combination of adhesive signals involving VAP-1 and CX_3_CR1-mediated integrin-activation and localized in areas of active inflammation and fibrosis in livers [28], these data support the concept that CD16^+^ monocytes/macrophages in IA patients play an important role in liver damage and fibrosis. Third, monocytes produced high levels of TNF, which are a major cytokine involved in several types of liver damage. Furthermore, the CD16^+^ subset of monocytes not only produced high levels of TNF, but also secreted a large amount of IL-6, IL-1β and IL-12/IL-23 P40. These pro-inflammatory cytokines which are critical for the process of liver damage during hepatitis B progression are also responsible for the differentiation of Th17 cells. All together, these data indicate that the CD16^+^ subset of monocytes may be closely related to liver damage in these IA patients.

Recent studies have demonstrated that activated monocytes/macrophages isolated from the site of inflammation may induce Th17 responses [19]–[21]. The present study further indicated monocytes/macrophages in LILs exhibited higher HLA-DR expression than peripheral monocytes. Furthermore, the CD16^+^ subset in LILs expressed higher levels of HLA-DR than those in the periphery. These findings indicated that monocytes with its subsets are highly activated under the inflammatory microenvironment in the liver. Indeed, our previous studies indicated that increased multiple pro-inflammatory cytokines, including IL-6, IL-1β and IL-12/IL-23 in the peripheral blood and liver, are present in CHB patients [6], [27]. Furthermore, increased IL-17 can directly affect monocytes and mDCs infiltrated into the liver, thereby exacerbating the inflammatory microenvironment of the liver. This cytokine milieu in the liver, including IL-1β, IL-6 and IL-23, may regulate the differentiation and expansion of human Th17 cells. Interestingly, we found that HBcAg can induce a profound effect on Th17 cells proliferation in the presence of these pro-inflammatory cytokines. These preliminary data indicated that the microenvironment in inflammatory liver, at least in part, may contribute to the differentiation and expansion of Th17 cells in these patients.

The present observations of the neutrophils were absolutely in line with the available literature on neutrophil biology in a mouse model of cholestatic liver damage [29], [30] and further indicated that the accumulated neutrophils in liver, as well as the decreased numbers of neutrophils in peripheral blood, are associated with liver injury in CHB patients. In addition, Th17 cells were shown to activate the IL-17 receptor-expressing cells in the liver, while the latter recruited neutrophils through IL-8 and growth related oncogen α secretion in alcoholic liver disease [23]. Furthermore, IL-8 levels in livers of CHB patients are increased [4]. Together with these findings, our study suggested that the accumulated Th17 cells in the liver, at least in part, may attract neutrophils. Future studies should investigate the mechanisms that govern neutrophil-induced liver damage in CHB patients.

Taken together, our data demonstrated, for the first time, that monocytes as well as pro-inflammatory CD16^+^monocytes and neutrophils participate in the modulation of inflammatory response and liver damage in CHB patients, and provide insight into the effect of these highly pathogenic cells on Th17 cells. These findings further demonstrate that targeting of inflammatory monocytes could lead not only to a decrease in the production of pro-inflammatory cytokines and Th17 cells, but also to the disruption of the cycle of inflammation due to induction and expansion of these cells.
